# Drosophila Hephaestus/Polypyrimidine Tract Binding Protein Is Required for Dorso-Ventral Patterning and Regulation of Signalling between the Germline and Soma

**DOI:** 10.1371/journal.pone.0069978

**Published:** 2013-07-23

**Authors:** Suzanne M. McDermott, Ilan Davis

**Affiliations:** Department of Biochemistry, University of Oxford, Oxford, United Kingdom; University of Dayton, United States of America

## Abstract

In the Drosophila oocyte, *gurken* (*grk*) mRNA encodes a secreted TGF-α signal that specifies the future embryonic dorso-ventral axes by altering the fate of the surrounding epithelial follicle cells. We previously identified a number of RNA binding proteins that associate specifically with the 64 nucleotide *grk* localization signal, including the Drosophila orthologue of polypyrimidine tract-binding protein (PTB), Hephaestus (Heph). To test whether Heph is required for correct *grk* mRNA or protein function, we used immunoprecipitation to validate the association of Heph with *grk* mRNA and characterized the *heph* mutant phenotype. We found that Heph is a component of *grk* mRNP complexes but *heph* germline clones show that Heph is not required for *grk* mRNA localization. Instead, we identify a novel function for Heph in the germline and show that it is required for proper Grk protein localization. Furthermore, we show that Heph is required in the oocyte for the correct organization of the actin cytoskeleton and dorsal appendage morphogenesis. Our results highlight a requirement for an mRNA binding protein in the localization of Grk protein, which is independent of mRNA localization, and we propose that Heph is required in the germline for efficient Grk signalling to the somatic follicle cells during dorso-ventral patterning.

## Introduction

The intracellular transport and subsequent localized translation of messenger RNA is a powerful and general strategy used to temporally and spatially restrict specific proteins to their site of function [1], [2], [3], [4], [5], [6]. Asymmetric protein distribution arising from the translation of localized mRNAs govern the regulation of cell polarity [7], [8], [9] and the segregation of cell fate and embryonic polarity determinants in oocytes and embryos [10], [11], [12]. In the developing *Drosophila* oocyte, the localization of *gurken* (*grk*), *oskar* (*osk*), *bicoid* (*bcd*) and *nanos* (*nos*) mRNAs plays a fundamental role in the determination of the axes of the oocyte and future embryo. *grk* encodes a TGF-α family signal [13] that acts as a secreted oocyte ligand for the *Drosophila* epidermal growth factor receptor (EGFR) [14], [15], [16]. Localization of *grk* mRNA and local translation of Grk at the dorso-anterior corner of the oocyte in mid-oogenesis [13], [17], [18] results in the localized activation of EGFR in the overlying follicle cells, inducing them to adopt a dorsal fate, thereby defining the dorsal-ventral axis of the egg and future embryo [19], [20]. Concomitant with *grk* localization to the future dorsal anterior region of the oocyte, *osk* mRNA accumulates at the posterior pole. Locally translated Osk protein nucleates the assembly of the germ plasm, which determines germ cell fate in the embryo [21], [22]. Osk-dependent assembly of germ plasm is also required for the posterior localization and translation of *nos* mRNA, which is in turn required for abdomen formation in the embryo [23], [24], [25].

The transport of mRNA cargoes to their cellular destinations occurs in large ribonucleoprotein (RNP) complexes termed transport particles or granules [17], [18], [26], [27], [28]. These complexes coordinate and control multiple steps including transport of the mRNA along the cytoskeleton by molecular motors, translational repression of the localizing mRNA, in addition to its anchoring and translational activation upon reaching the final destination [4]. The composition, organization and dynamics of such complexes therefore dictate the behaviour and fate of the target RNA in the cell. However, many questions remain unanswered about these transport RNPs and their association with localized mRNAs. Although many of the known proteins that function in *grk* mRNA localization and translational regulation have been identified through genetic studies [13], [29], [30], [31], [32], [33], we recently identified, using complementary biochemical approaches, new proteins that could potentially function in *grk* regulation. We used GRNA chromatography to identify proteins that can associate specifically with the *grk* localization signal (GLS) [34], a 64-nucleotide stem loop structure in the coding region of the *grk* transcript that is necessary and sufficient for dorso-anterior localization of the RNA [35]. We identified a number of known factors previously shown to be required for *grk* mRNA localization and translational regulation [13], [29], [30], [31], [33] as well as a number of other RNA binding proteins that were not previously implicated in the function of *grk.* These included Syncrip, which we showed was required for grk mRNA localization and translational regulation, and the *Drosophila* orthologue of polypyrimidine tract-binding protein (PTB), Hephaestus (Heph), which was previously only known to play a role in *osk* mRNA localization.

Polypyrimidine Tract Binding (PTB) protein is an hnRNP family member that is thought to act as an RNA chaperone with the ability to bind RNA in a sequence specific manner and to restructure the RNA substrate [36], [37], [38], [39], [40], [41]. It acts to regulate a number of forms of RNA metabolism, including negative and positive regulation of alternative splicing [39], [40], [42], [43], [44], [45], [46], 3′-end processing [47], stabilization [48], miRNA-mediated gene regulation [49], translation [50], [51], [52], [53], and, of particular relevance to this work, mRNA localization and localized translation [54], [55], [56], [57], [58]. *Drosophila* PTB is encoded by the *hephaestus (heph)* locus, and shares over 50% amino acid identity and 60% similarity with vertebrate forms of PTB (mammalian PTB/hnRNPI and Xenopus VgRBP60). Genetic screens and developmental studies indicate that *Drosophila* Heph/PTB regulates spermatogenesis in adult males [59], [60], [61], [62], embryonic development [63] and sensory bristle and wing margin development during larval and pupal stages [64], [65], [66]. Work by Besse et al. [55] identified Heph as a key structural component of *osk* RNP complexes in the *Drosophila* oocyte, required for efficient *osk* mRNA localization and also translational repression of *osk* mRNA during localization. Heph is required indirectly for *osk* localization through the properly timed organization of the oocyte microtubule (MT) cytoskeleton, but directly controls Osk translation through the multimerization of individual *osk* mRNA molecules and the formation of high order translationally repressed *osk* RNP complexes.

As Heph associates with *grk* and GLS RNA in vitro we set out to test the hypothesis that Heph is a component of a *grk* RNP at one or a number of stages of oogenesis and plays a role in *grk* localization, anchoring, or localized Grk translation. We show using RNA immunoprecipitation (RNA IP) and analysis of *heph* mutant germline clones that Heph is a component of *grk* mRNP complexes but is not required for *grk* mRNA localization. Instead, Heph is required in the germline for proper Grk protein dorso-anterior localization and for efficient Grk signalling from the germline to the somatic follicle cells.

## Results

### Heph is a Component of *grk* RNP Complexes

**Figure 1:**
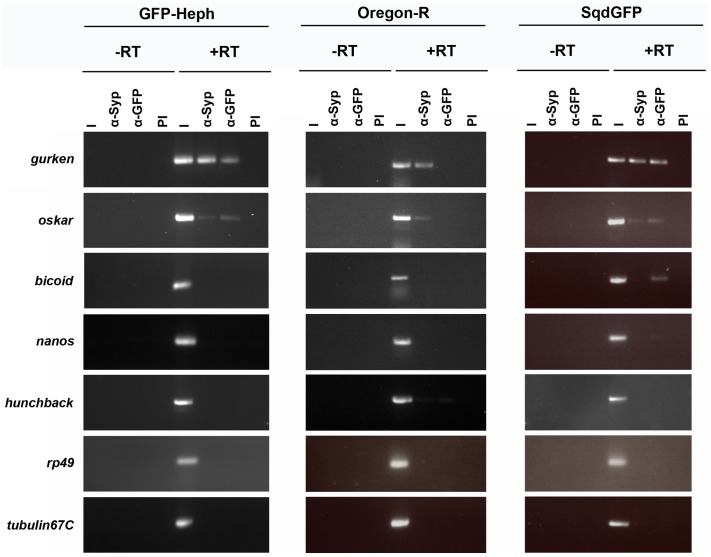
Heph associates with *grk* and *osk* mRNAs. RT-PCR amplification of mRNAs recovered in fractions immunoprecipitated from GFP-Heph protein trap, SqdGFP control or Oregon-R control ovarian extracts using anti-GFP and control anti-Syp or pre-immune rabbit serum. I = Input RNA (10%); PI = control rabbit serum; −/+RT = minus/plus reverse transcriptase.

GRNA chromatography studies have shown that Heph associates in vitro with *grk,* and more specifically, the GLS (Table S1) [34]. It has also been previously reported that Heph/PTB represses the translation of *osk* by promoting the formation of large, high-order *osk* containing messenger ribonucleoprotein (mRNP) complexes [55]. To ask whether Heph is a component of *grk* RNPs in the germline, we carried out RNA IP experiments using anti-GFP antibodies and previously characterized GFP-PTB/Heph protein-trap ovaries [55]. RT-PCR showed that *grk* and *osk* mRNAs are present in the fractions precipitated from GFP-PTB/Heph protein-trap ovaries and from SqdGFP control ovaries using anti-GFP and control anti-Syncrip antibodies, but not in immunoprecipitates using control rabbit serum, or in anti-GFP immunoprecipitates from wild-type control females (Figure 1). No specific enrichment of highly expressed *rp49* and *tubulin67C* mRNAs, of the localized *bcd* and *nos* mRNAs, or of the non-localized hunchback mRNA control from the initial GRNA chromatography experiments [34] was observed in the GFP-PTB/Heph precipitated fractions. These results, combined with the earlier GRNA results [34] indicate that Heph is a component of *grk* mRNP complexes in vivo.

To test where Heph protein is expressed in oogenesis we stained wild type egg chambers with anti-PTB/Heph antibodies. We found that the protein is localized in the cytoplasm of the follicular epithelial cells throughout oogenesis (Figure S1A–E). In the germline, Heph is present throughout the oocyte and nurse cell cytoplasm with an accumulation at the posterior pole of the oocyte from around stage 9 of oogenesis onward (Figure S1C–E). These signals are reduced in *heph^e1^* hypomorphic mutant germline clones (Figure S1H and I), confirming that the protein detected in wild-type egg chambers is indeed specifically Heph. The posterior localization of Heph is particularly strong in late stage 10 egg chambers (Figure S1D–E), and reflects Heph’s role as a key structural component of *osk* mRNP complexes [55]. Despite a general signal throughout the oocyte cytoplasm, Heph does not accumulate at the dorso-anterior cap and so does not colocalize with localized *grk* mRNA (Figure S1C–G). This indicates that the interaction of Heph with *grk* mRNA may be transient and occur at earlier stages of *grk* localization before the RNA reaches the dorso-anterior cap.

The pattern of localization observed here is consistent with that of both endogenous Heph and GFP-PTB/Heph previously shown by Besse et al. [55]. In these previous studies endogenous Heph and GFP-PTB/Heph were also additionally present in the nuclei of both the nurse cells and the follicular epithelial cells throughout oogenesis, and may not have been seen here due to the use of different antibodies or conditions for immunostaining. However, in both studies it is clear that there is no enrichment of Heph at the dorso-anterior cap.

### *heph* Mutant Eggs have Patterning and Morphological Defects

To investigate the role of Heph in *grk* mRNA regulation, three different alleles [55],[64] belonging to the same lethal complementation group were used (Table 1). These alleles also fail to complement the male sterility of the original P-element-induced allele *heph^2^* [59]. *heph^03429^* is a P-element insertion that maps to a large intron. *heph^e2^* is an EMS-induced mis-sense mutation that behaves as a null allele and corresponds to a deletion covering RRM 1, RRM 2 and part of RRM 3, whilst *heph^e1^* substitutes a conserved glycine (G) residue to a glutamine (Q) residue in RRM 1 [64]. *heph^03429^* and *heph^e1^* are hypomorphic alleles that strongly affect Heph expression [55]. Due to the lethality of these alleles, it was necessary to make germline clones using the FLP-dominant female sterile (DFS) technique [67],[68]. As previously observed [55], germ cells homozygous for *heph^e2^* fail to produce differentiated egg chambers, suggesting that Heph function is required for early germ cell maintenance, whilst germ cells homozygous for *heph^03429^* and *heph^e1^* do support development until later stages of oogenesis.

**Table 1:** Alleles associated with the heph locus.

allele	mutagen	mutation	viable	complementation deficiency DfG45	complementation heph*^03429^*	origin
*heph^e2^*	EMS	deletion of RRMs 1, 2 and part of 3	no	no	no	Dansereau et al. [64]
*heph^e1^*	EMS	point mutation (G to Q in RRM1)	no	no	no	Dansereau et al. [64]
*heph^03429^*	P-element	insertion at position 27756259 (intron)	no	no	–	Besse et al. [55]

A number of alleles map to the *heph* locus. Two EMS-induced alleles, *heph^e1^* and *heph^e2^*, alter RRM domains. *heph^e2^* is a deletion of several coding exons including the coding region for RRM1, RRM2 and part of RRM3. *heph^e1^* is a mis-sense mutation that changes a glycine (G) to a glutamine (Q) in RRM1. The P-element allele *heph^03429^* [64] maps to a *heph* intron.

doi:10.1371/journal.pone.0069978.t001

**Figure 2:**
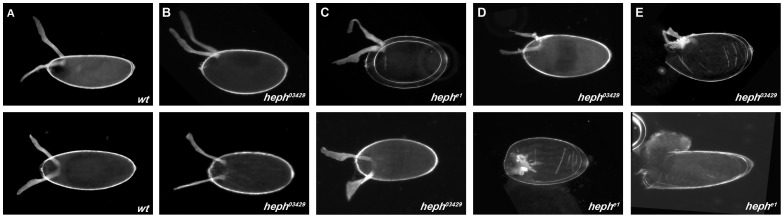
*heph* mutant eggs have patterning defects. (**A**) Wild-type egg with two dorsal appendages that mark the dorsal anterior surface. (**B–D**) Eggs are shorter and rounder in the *heph* mutant alleles and have a range of dorsal appendage defects from (**B**) spacing defects (**C**) broad with ragged edges to (**D**) shorter with ‘antlers’, joined by additional dorsal appendage material. (**E**) A small number of eggs are fully open at the anterior. See Table 2 for quantitation of eggshell phenotypes.

Local translation of Grk at the dorso-anterior of the oocyte is required for dorso-ventral axis determination in both the eggshell and the embryo. Grk signalling causes the overlying follicle cells to adopt a dorsal cell fate, allowing at a later stage for the correct specification and positioning of anterior and dorsal chorion structures, such as the dorsal appendages. Analysis of patterning and dorsal appendage morphogenesis in the eggshell is therefore a sensitive way in which to investigate *grk* localization, Grk translation and Grk signalling within the egg chamber. Analysis of *heph* mutant eggs revealed a number of eggshell phenotypes. None of the eggs laid were fully wild-type (class A) (Figure 2A–E and Table 2), and most were shorter and rounder than wild-type eggs (class B–D) (Figure 2B–D, and Table 2). There were a number of phenotypes seen in the dorsal appendages; wild-type length and morphology (class B), broader than wild-type with ragged edges (class C), shorter with ragged edges, resembling ‘antlers’, joined at the centre by additional dorsal appendage material (class D). Within class B and C, a number of eggs were observed where inter-dorsal appendage spacing was greater than that observed in wild-type eggs. A small number of eggs were also fully open at the anterior (class E), resembling eggs laid by *cup* mutants. The distribution of the numbers of eggs in the different classes varies for *heph^e1^* and *heph^03429^* eggs. The majority of *heph^e1^* eggs were class D, whilst *heph^03429^* eggs were class B or C, suggesting that *heph^e1^* mutant eggs have a slightly more severe phenotype, and is probably a reflection of the difference in the molecular nature of the two alleles.

**Table 2:** Quantitation of eggshell phenotypes observed for *heph^e1^* and *heph^03429^* germline clones.

Genotype	A (wild-type)	B	C	D	E
OrR (n = 153)	100	0	0	0	0
*heph^e1^* (n = 111)	0	24.3	10.8	56.8	8.1
*heph^03429^* (n = 135)	0	42.2	42.2	8.9	6.7

Numbers are given as percentages of the total number of eggs counted. Oregon R (OrR) served as a wild-type control strain.

doi:10.1371/journal.pone.0069978.t002

The eggshells shown in Figure 2B–D have characteristics of those with dorso-ventral patterning defects. The eggs are short and round, the dorsal appendages are broader than wild-type, there is an increase in the inter-appendage space between the two dorsal appendages, and there is ectopic dorsal appendage material at the dorsal midline. The dorsal appendages of these eggs also resemble the ‘moose antlers’ seen in eggs that develop from *bullwinkle* (*bwk*) mutant germline clone egg chambers [69],[70], where the follicle cell migrations that form the dorsal appendages are abnormal. The lack of wild-type *heph* function in the germline also results in a number of phenotypes that may reflect defects in the germline actin cytoskeleton. The eggs resemble the short eggs seen for mutants such as *chickadee* [71] and *quail* [72], where actin dynamics or regulation is perturbed. Staining of *heph* mutant germline clones with phalloidin shows that the dense bundles of actin filaments that ordinarily form in the nurse cell cytoplasm at stage 10, prior to dumping at stage 11, do not form in a small number of *heph* mutant germline clones (Figure S2), and aberrant actin patches are also observed in the oocyte cytoplasm (Figure S2B). It is unclear whether these actin patches are free-floating, detached ring canals, or the accumulation of actin spheres. Such actin spheres are observed in *kinesin heavy chain* (*khc*) [73] and also *Bic-C* mutant egg chambers [74]. A partial dumpless phenotype (due to incomplete transfer of nurse cell contents into the oocyte at stage 11) is also observed. The eggshells shown in Figure 2E are fully open at the anterior, and resemble the eggs laid by *cup* mutant females. Such defects may result from insufficient oocyte growth and the subsequent misalignment of the follicle cell migration that would cover the anterior of the oocyte (for review of eggshell patterning see [75]). In summary, examination of the eggshells of *heph* germline clones reveals multiple roles for *heph* in the female germline, ranging from regulation of dorso-ventral patterning and correct migration of the somatic follicle cells, to organization of the actin cytoskeleton in the germline.

### *grk* mRNA Localization is not Perturbed in *heph* Mutant Oocytes

**Figure 3:**
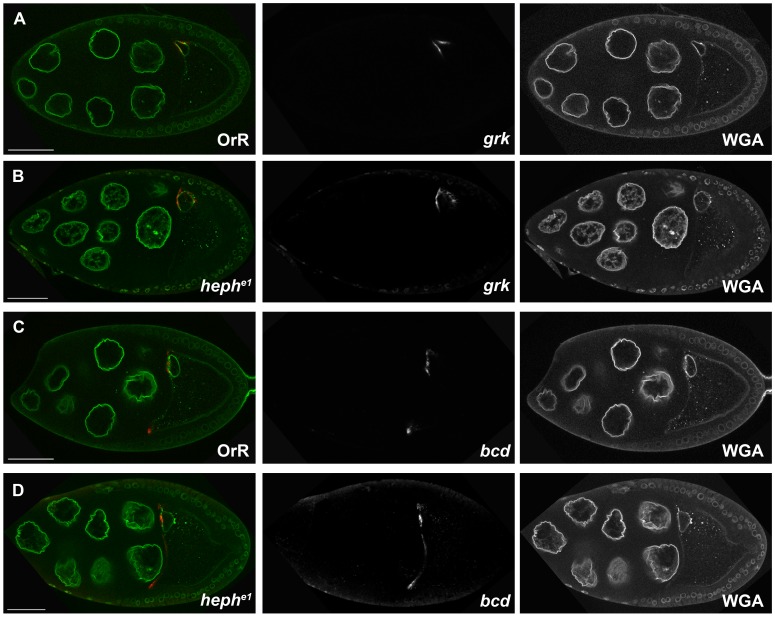
*grk* mRNA localization is not perturbed in *heph* mutant oocytes. *In situ* hybridization using a *grk* or *bcd* probe was carried out on Oregon-R (OrR) (**A**) and (**C**)**,** or *heph^e1^* germline clone egg chambers (**B**) and (**D**). Egg chambers were also stained with fluorescent Wheat Germ Agglutinin (WGA) conjugate in order to label nuclear membranes. Scale bar 50 µm.

In an effort to understand the nature of the dorso-ventral patterning defects seen in *heph* mutant eggs, and the presence of Heph in *grk* mRNP complexes, we investigated the distribution of *grk* mRNA in *heph* mutant egg chambers. In wild-type egg chambers, the pattern of *grk* mRNA localization changes during the progression of oogenesis. In early oogenesis (stages 1–7), *grk* is localized in a posterior crescent between the nucleus and the overlying follicle cells. During stages 7 and 8, *grk* begins to accumulate around the new dorso-anterior position of the nucleus as well as forming a transient ring at the anterior of the oocyte. From stages 9 to 10B, *grk* is tightly localized in a dorso-anterior cap near the oocyte nucleus (Figure 3A and Figure S3). *grk* mRNA localization in *heph^e1^* and *heph^03429^* oocytes was analysed by *in situ* hybridization and found to be identical to the wild-type pattern of localization at all stages of oogenesis (shown for *heph^e1^* oocytes Figure 3B and Figure S3). Fluorescently labelled full-length *grk* RNA also localized to the dorso-anterior cap when injected into *heph^e1^* and *heph^03429^* stage 9 oocytes in an in vivo injection assay [18], indicating that Heph is not required in the oocyte cytoplasm for *grk* localization (data not shown). *bcd* localization was also unaffected in *heph* mutant germline clones (Figure 3C and D). This was to be expected as RNA IP experiments (Figure 1) indicate that Heph is not a component of *bcd* mRNP complexes.

### Grk Protein is Mislocalized in *heph* Mutant Oocytes

**Figure 4:**
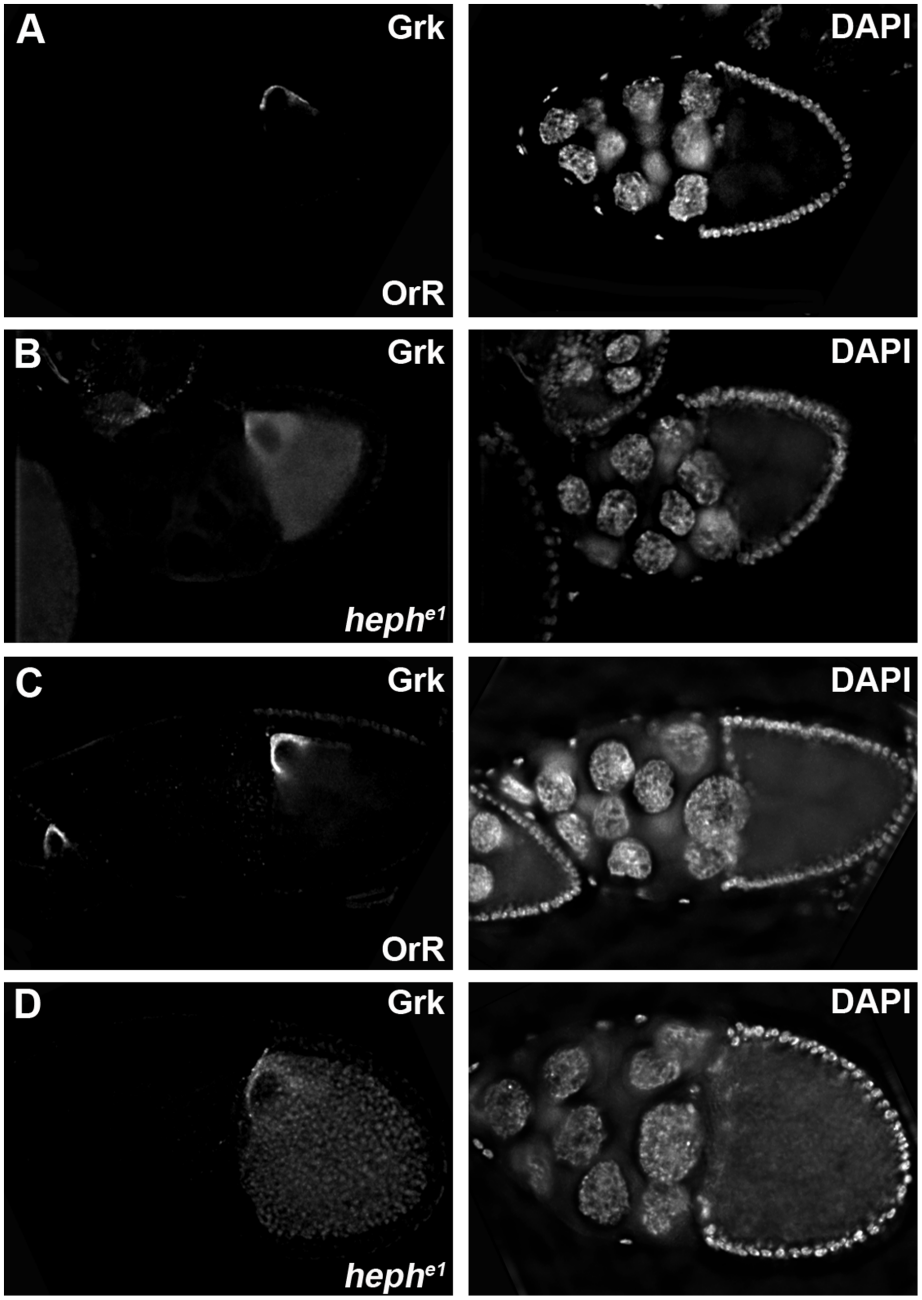
Grk protein is mislocalized in *heph* mutant stage 10 oocytes. Stage 10A (**A**) and stage 10B (**C**) Oregon-R (OrR) egg chambers, and stage 10A (**B**), and stage 10B (**D**) *heph^e1^* germline clone egg chambers were stained with anti-Gurken and DAPI. In *heph^e1^* germline mutant egg chambers at stages 10A and 10B Grk protein is evenly distributed throughout the oocyte.

Next we investigated the distribution of Grk protein. Interestingly, in contrast to the normal localization of *grk* mRNA, we found an abnormal distribution of Grk protein in *heph* germline clones. During early and mid-oogenesis the distribution of Grk protein mirrors that of *grk* mRNA, reflecting the two signalling events in which Grk acts to polarize both the follicular epithelium and the future embryo. At stage 6, Grk is enriched at the posterior of the oocyte (Figure S4A), whilst by stage 9 Grk is at the dorso-anterior cap (Figure S4C). By the later stages of oogenesis (stages 10–12) the distribution of Grk protein extends over approximately half the length of the dorsal midline of the oocyte to form an elongated anterior to posterior stripe [76] (Figure 4A and C) where Grk is present in the oocyte cytoplasm close to the nucleus, in neighbouring follicle cells, and in the extracellular space between the cortices of the oocyte and the follicular epithelium [77].

In *heph* mutants, posterior Grk localization and signalling at stage 7 of oogenesis is normal as the nucleus migrates correctly in all *heph* mutant oocytes. Grk protein localization is also generally normal up to stage 9 of oogenesis (Figure S4), although in a small number of mutant oocytes we see mild defects where Grk protein is present throughout the oocyte cytoplasm in addition to being at the dorso-anterior (Figure S4E and F). In stage 10A and 10B *heph* mutant oocytes this mislocalization of Grk in the oocyte cytoplasm is more pronounced and evident in large proportions of *heph* mutant oocytes (Figure 4B and D). In 42.9% *heph^03429^* stage 10A (n = 35) oocytes and 66.7% of 10B (n = 36) oocytes Grk was dispersed throughout the oocyte cytoplasm. The percentage of oocytes with this defect was higher in the *heph^e1^* mutant where 96.9% stage 10A (n = 32) and 97.3% stage 10B (n = 37) oocytes showed Grk throughout the oocyte cytoplasm (Figure 4B and D). The fact that the total levels of Grk protein are unaffected in *heph* mutant ovaries (Figure S5), leads us to conclude that the phenotype is due to mislocalization of Grk protein, and not to changes in the expression level of Grk. This mislocalization is not a result of its mRNA mislocalization, indicating that Heph is having a specific effect on Grk protein localization.

### BR-C Protein is Expressed in all Follicle Cells in *heph* Mutant Stage 10 Egg Chambers

**Figure 5:**
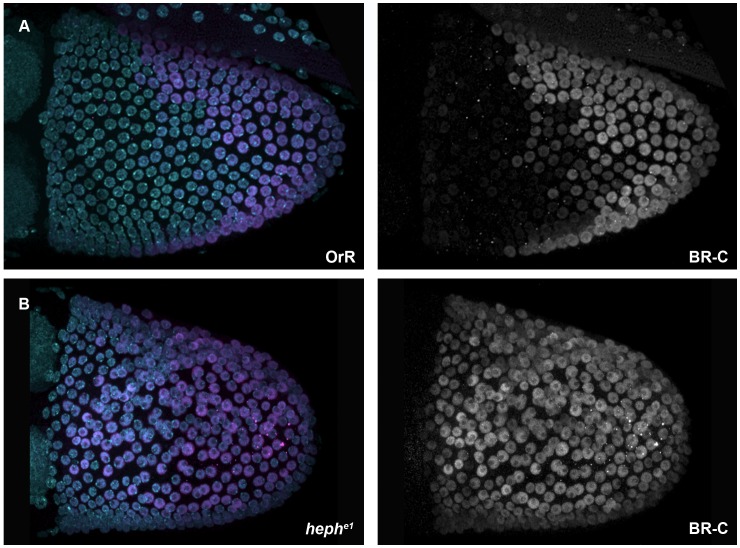
BR-C protein is expressed in all follicle cells in *heph* mutant stage 10 egg chambers. Stage 10 Oregon-R (OrR) egg chambers (**A**) and *heph^e1^* germline clone egg chambers (**B**) were stained with anti-Broad-core and DAPI. In stage 10 OrR egg chambers, BR-C is not detected in the follicle cells at the dorsal anterior region (the ‘dorsal gap’) (**A**), whilst in *heph^e1^* germline mutant egg chambers BR-C is detected in all dorsal follicle cells (**B**).

The mislocalization of Grk protein could potentially be due to defects in Grk cleavage or secretion from the oocyte. To visualize and assess whether Grk can be secreted and therefore signal to the neighbouring follicle cells in *heph* mutant germline clones, we examined the expression of the *Broad-Complex* (*BR-C*) locus. *BR-C* encodes a group of four alternatively spliced zinc finger transcription factor isoforms whose elevated expression from stage 10 of oogenesis marks the follicle cells that will later form the dorsal appendages [78]. Analysis of *BR-C* expression in multiple mutants supports a mechanism whereby *BR-C* is induced above a critical level of EGFR activation, but is repressed at a higher level [79],[80]. This repression results in a lack of BR-C staining at the dorsal midline (the ‘dorsal gap’) that receive the highest level of Grk signal (Figure 5A) and ultimately in the formation of two separate dorsal appendages. In approximately 63% (n = 11) *heph^e1^* germline mutant egg chambers BR-C is detected in all dorsal follicle cells, and there is no dorsal gap (Figure 5B). This is consistent with a number of the observed eggshell phenotypes including changes in the inter-appendage space between the two dorsal appendages, the presence of ectopic dorsal appendage material at the dorsal midline and defects in dorsal appendage morphogenesis. It is also consistent with the defects in Grk signalling in these egg chambers, and could indicate that follicle cells in the dorsal midline are not receiving the high levels of Grk signalling as they would in wild-type egg chambers, but are receiving an intermediate level of signalling normally seen by cells either side of the midline.

## Discussion

We identified Heph as a factor able to associate in vitro with *grk* and GLS-containing RNA [34] leading us to hypothesize that it is a trans-acting factor required for the localization, anchoring or translational control of *grk* mRNA in the *Drosophila* oocyte. This hypothesis was supported indirectly by the fact that a function for the Heph/PTB in the formation of high-order *osk* mRNP complexes and the subsequent translational repression of *osk* in the oocyte was also previously characterized [55]. Using RNA IP experiments we have shown here that Heph binds specifically to *grk* and *osk* mRNAs. These results are consistent with the previous in vitro RNA-pulldown experiments [34] and therefore suggest that Heph is a component of the *grk* mRNP complexes, in addition to complexes containing *osk* [55]. Heph does not colocalize with *grk* mRNA at the dorso-anterior corner of the oocyte, suggesting that this interaction occurs at earlier stages of *grk* localization before the RNA reaches its final destination, and may also be transient. This is in contrast to the case of *osk*, where Heph/PTB can be visualized at the posterior pole where it colocalizes with *osk* mRNA [55].

Our examination of *heph* mutant germline clones revealed multiple functions for *heph* during oogenesis in addition to the previously characterized roles of Heph in early germ cell maintenance and *osk* translational repression [55]. Follicle cell phenotypes observed in mutant germline clones indicate that Heph may be involved in signalling between the germline and the somatic follicle cells, and in the subsequent patterning and morphogenesis of the dorsal appendages. We have shown that Heph is not required for *grk* mRNA localization, despite the interaction of Heph with *grk* mRNP complexes. Nor is Heph required for Grk protein localization and signalling in the earlier stages of oogenesis. However, Heph is required for correct Grk protein localization and Grk signalling in stage 10 oocytes as assessed by Grk and BR-C expression.

It remains unclear why loss of *heph* function would result in the mislocalization of Grk protein during stage 10 of oogenesis when *grk* mRNA localization is unaffected. The dispersal of Grk throughout the oocyte in *heph* mutants suggests that Heph could function in the trafficking of Grk protein after translation [74],[77],[81],[82],[83],[84],[85]. There are a number of previously studied mutants, such as *rab6* [83],[86], and *syntaxin 1A* (*Syx1A*) [84] in which *grk* mRNA localization is unaffected, but Grk protein is mislocalized. Furthermore, in these mutants, as is the case with *heph*, only the dorso-anterior localization and signalling of Grk protein is affected, and not the posterior localization or signalling during earlier stages of oogenesis. This is thought to be a consequence of a lower threshold for EGFR activation by Grk signalling in the posterior follicle cells compared with the dorso-anterior follicle cells [74]. In null germline clones of the small GTPase Rab6, Grk mislocalizes to droplet-like structures throughout the ooplasm [83],[84] that colocalize with post-Golgi vesicle markers [83]. In hypomorphic germline clones of the Syx1A t-SNARE protein, Grk protein is also diffused throughout the ooplasm and partially colocalizes with Rab6 [84]. Localization of Grk protein at the dorso-anterior of the oocyte therefore depends upon membrane trafficking, and it is possible that Heph also functions to regulate and restrict Grk protein trafficking only to the dorso-anterior corner of the oocyte. With reduced Heph activity, follicle cells other than those at the dorso-anterior corner would then receive ectopic low-level Grk signalling which could explain the BR-C expression pattern and eggshell phenotypes that we observed.

The underlying molecular cause for a Grk mislocalization or trafficking defect in *heph* mutants is also uncertain. It is possible that Heph directly regulates the expression of mRNAs encoding proteins that are required to act in the secretory pathway, such as Rab6 and Syx1A. Vesicular trafficking is also coupled with actin cytoskeletal dynamics [87],[88], and it is possible that Grk mislocalization or trafficking defects could be due to defects in the actin cytoskeleton. The lack of wild-type *heph* function in the female germline results in egg chamber and eggshell phenotypes consistent with defects in actin regulation. The short eggs have a partial dumpless phenotype that is presumably caused by the lack of the dense bundles of actin filaments that ordinarily form in the nurse cell cytoplasm prior to dumping. This phenotype is seen in a small number of mutant germline clones, but it is possible that a higher number of clones have more subtle defects in actin filament organization that were missed in our analysis. Actin regulation by Heph has also been observed in *Drosophila* spermatogenesis [61], where loss of a male specific Heph isoform in the testes results in disruption of the actin cones of the spermatid individualization complex and prevents proper individualization of spermatids. Phenotypes seen in *heph* mutants during embryonic development can also be attributed to defects in actin regulation [63]. *heph* mutant embryos are defective in dorsal closure, and actin accumulates to high levels in a disorganized fashion in dorso-lateral regions. Whether Heph directly acts on mRNAs that encode cytoskeletal components is unknown. Indeed, mammalian PTB regulates members of the actin cytoskeleton, including α- and β-tropomyosin and α-actinin [89],[90].

There is a precedent for an RNA-binding protein such as Heph to regulate actin organization and vesicle trafficking within the oocyte. Bicaudal-C (Bic-C) is a KH-type RNA-binding protein that is also required for translational repression of unlocalized *osk* [91]. In *Bic-C* mutant egg chambers, Grk is mislocalized during mid-oogenesis and sequestered within actin-coated structures that contain distinct sets of secretory proteins in addition to microfilament interacting proteins [74],[92]. Bic-C is thought to affect the expression of a number of proteins during oogenesis and several Bic-C RNA targets encode proteins that are predicted to be involved in vesicle trafficking and/or organization of the actin-cytoskeleton [93]. Interestingly, like Heph, Bic-C was also found to associate in vitro with *grk* in GRNA chromatography studies (Supplementary Table 1) [34], although analysis of hatching frequency and viability of progeny from trans-heterozygotes of *Bic-C* and *heph* mutant alleles do not reveal any genetic interaction between *Bic-C* and *heph* (data not shown).

Our results highlight several perhaps unexpected issues. First, it is likely that many more factors are required for various aspects of *grk* mRNA localization, translational regulation and Grk signalling, including previously identified factors as well as unknown ones. Second, it is clear from our work that additional genetics studies are required to characterize the factors identified biochemically as binding to localized transcripts in the oocyte. Third, there are several aspects of Heph function in oogenesis that are not fully understood. In order to elucidate the primary functions of Heph in the germline, and the observed *heph* mutant phenotypes, future experiments will have to uncover the RNA targets for Heph. This will provide insight into the pathways that are perturbed in *heph* mutants and may well discover some unexpected mechanisms that could explain the complexity of the *heph* phenotype. For example, Heph is also required indirectly for *osk* localization through the properly timed organization of the oocyte microtubule (MT) cytoskeleton, and in *heph* mutants there is a delay in focusing the microtubule plus-ends at the posterior pole of the oocyte [55]. It is possible that this defect in the efficient establishment of microtubule polarity in the oocyte is a consequence of defects in actin organization [94],[95] and/or vesicular trafficking [86], or could indicate a more direct role in regulating the expression of proteins that organize the microtubule cytoskeleton. Finally, one particularly intriguing question relates to why Heph is present in *grk* mRNP complexes if it is a regulator of trafficking and/or of the actin cytoskeleton as we hypothesize. As a component of the *grk* RNP, it is possible that Heph could act at the interface of *grk* localization and the secretory pathway in order to promote efficient and restricted local secretion of Grk protein, providing an example of how interaction between secretory and mRNA processing pathways can promote efficient protein trafficking [74],[85].

## Materials And Methods

### Genetics

Stocks were raised on standard cornmeal-agar medium at 25°C. The wild-type control strain was Oregon R (OrR). The P(PZ}heph[03429] (Bloomington Stock Center) had been recombined onto the FRT82B chromosome by Besse et al. [55], and was a gift from A. Ephrussi. The FRT82B-recombined EMS mutant alleles heph^e1^ and heph^e2^ were characterized by Dansereau et al. [64] and were a gift from W. Brook. Germline clones of heph were generated using the FLP/FRT dominant female sterile technique [67],[68]. Females heterozygous for the FRT82B-recombined *heph^e1^*, *heph^e2^* and *heph^03429^* alleles were mated to P{ry^+^, FRT}82B P{w^+^ovoD1}/TM3 sb males and the progeny third instar larvae were heat shocked at 37°C for 2 h each day for 3 days. *heph^e1^* and *heph^03429^/ovo^D1^* females were collected and mated to OrR males. GFP-PTB/Heph was a GFP protein trap line [55] and was a gift from A. Ephrussi. SquidGFP was also a GFP protein trap line from A. Debec.

### Eggshell Preparation

Axis specification defects were studied in eggs from *heph* mutant germline clones. For eggshell preparation, freshly laid eggs were mounted in a 1∶1 mixture of lactic acid:Hoyer’s medium and incubated overnight at 65°C.

### Immunoprecipitation

Rabbit anti-GFP (Invitrogen), rabbit anti-Syp (S. McDermott, unpublished), and control rabbit pre-immune sera were cross-linked to Protein A Sepharose using standard protocols, and the beads equilibrated in IP buffer (50 mM Tris-HCl pH 8.0, 150 mM NaCl, 0.5% NP-40, 10% glycerol and Complete EDTA-free protease inhibitor). For RNA immunoprecipitation all steps were carried out in the presence of RNasin (Promega). 20 µl of a 50% slurry was added to 500 µg precleared ovarian lysate, and the mixture incubated overnight at 4°C with mixing. The beads were then rinsed once with cold IP buffer and washed four times for 5 min each at 4°C. After the final wash, immunocomplexes were eluted from the beads by the addition of 100 µl extraction buffer (50 mM Tris-HCl pH 8.0, 10 mM EDTA and 1.3% SDS) plus 100 U RNasin and incubation for 30 min at 65°C. Following centrifugation, the elution step was repeated and the two supernatants combined. RNA was extracted from input samples and immunoprecipitates using Trizol LS reagent (Invitrogen). Following DNase (Ambion) treatment, immunoprecipitated RNA and 10% total RNA were then used as a template for cDNA synthesis in combination with SuperScript III (Invitrogen) RT and random hexamer primers (Invitrogen). cDNA was then used directly as a template for PCR with primers as described in Supporting Information.

### Western Blotting

30 to 50 µg of cleared ovarian lysate was loaded per well and ran on NuPAGE Novex 4–12% Bis–Tris Gels (Invitrogen). Following electrophoresis, proteins were transferred to Polyvinylidene Fluoride (PVDF) (Bio-Rad) membranes using the XCell II Blot Module (Invitrogen) according to the manufacturer’s instructions. Western blotting was performed using standard protocols. The mouse anti-Grk mAb1D12 concentrate (Developmental Studies Hybridoma Bank) was used at 1∶5000 and the rabbit anti-tubulin (Sigma) at 1∶2000. Rat anti-PTB/Heph was a gift from A. Ephrussi and was used at 1∶2000. Immunoreactive bands were visualized with HRP-conjugated sheep anti-mouse, and rat and donkey anti-rabbit secondary antibodies (GE Healthcare Life Sciences) at 1∶5000 and with enhanced chemiluminescence (GE Healthcare Life Sciences).

### Immunohistochemistry and in situ Hybridization

Ovaries were prepared for immunostaining as previously described (MacDougall et al., 2003). The following primary antibodies were used: mouse anti-Broad-core supernatant mAb25E9.D7 (1∶100) (Developmental Studies Hybridoma Bank), mouse anti-Grk mAb1D12 concentrate (1∶300) (Developmental Studies Hybridoma Bank) and rabbit anti-PTB/Heph (1∶500) (gift from A. Ephrussi). Alexa-conjugated secondary goat antibodies (1∶500) (Invitrogen) were used. Ovaries were also stained with phalloidin to label F-actin (Invitrogen) and with 10 µg/mL DAPI to label nuclei. In situ hybridization was performed as previously described (Wilkie et al., 1999). Digoxigenin-labeled antisense probes corresponding to either gurken (T. Schüpbach) or bicoid (C. Nüsslein-Volhard) full-length coding sequences were detected using sheep HRP-conjugated anti-DIG antibody (1∶500; Roche) followed by fluorescent tyramide signal amplification (Perkin Elmer). Ovaries were also stained with a fluorescent Wheat Germ Agglutinin conjugate (1 µg/mL) (Invitrogen) to label nuclear membranes.

### Synthesis of Fluorescently Labeled Capped RNA and Injection

RNA was transcribed in vitro using UTP-Alexa 546 or UTP-Cy3 (Wilkie and Davis, 2001) and a plasmid containing the full-length grk cDNA (T. Schupbach). Ovaries were dissected in Series 95 halocarbon oil (KMZ Chemicals) and egg chambers adhered to coverslips using tungsten needles. Fluorescently labeled RNA (250–500 ng/ml) was injected into oocytes using Femtotip needles (Eppendorf). At least two batches of RNA were injected for each experiment on separate occasions.

### Imaging and Deconvolution

A Leica SP5 confocal microscope and a widefield DeltaVision microscope (Applied Precision, Olympus IX70, and Roper Coolsnap HQ) were used to image fixed material. DeltaVision images were acquired with Olympus 20x/0.75, 40x/0.95, 60x/0.9 or 100x/1.4 objective lenses and then deconvolved.

## Supporting Information

Figure S1 **Distribution of Heph during** ***Drosophila*** **oogenesis.** Stages 4–6 (**A**), stage 8 (**B**), stage 9 (**C**) and stage 10B **(D and E)** egg chambers stained with anti-Heph antibodies and DAPI. (**E**) is an enlargement of the oocyte shown in the egg chamber in (D). (**F**) is an enlargement of the dorso-anterior corner (white box) of the stage 9 oocyte in (C) whilst (**G**) is an enlargement of the dorso-anterior corner (white box) of the stage 10B oocyte in (D). Heph is detected in the cytoplasm of the follicular epithelial cells throughout oogenesis. In the germline, Heph is present throughout the oocyte cytoplasm with an accumulation at the posterior pole of the oocyte from around stage 9 of oogenesis onward (C–E). However, Heph does not accumulate at the dorso-anterior cap coincident with *grk* mRNA (C–G). Scale bars in A, B, and E represent 60 µm. Scale bars in C and D represent 90 µm. (**H**) is the same wild-type egg chamber shown in (D) for comparison with (**I**), which is a *heph^e1^* germline clone egg chamber also stained with anti-Heph antibodies. Oocyte and nurse cell signals are lost in *heph^e1^* mutant egg chambers, indicating that the signal is specific.
(TIF)

Figure S2 **Actin structures are disrupted in** ***heph^03429^*** **and** ***heph^e1^*** **germline clone egg chambers.** Wild-type Oregon-R (OrR) (**A**), *heph^03429^* germline clone (**B**), and *heph^e1^* germline clone egg chambers (**C**) were stained with Phalloidin to label F-actin.
(TIF)

Figure S3 ***grk*** **mRNA localization is not perturbed in stage 10** ***heph*** **mutant oocytes.** *In situ* hybridization using a *grk* was carried out on Oregon-R (OrR) (**A**)**,** or *heph^e1^* germline clone egg chambers (**B**). Egg chambers were also stained with DAPI to label DNA.
(TIF)

Figure S4 **Grk protein localization in** ***heph*** **mutant stage 6 to stage 9 oocytes.** Stage 6 (**A**), stage 8 (**B**), and stage 9 (**C**) wild-type Oregon-R (OrR) egg chambers, and stage 6 (**D**), stage 8 (**E**), and stage 9 (**F**) *heph^e1^* germline clone egg chambers were stained with anti-Gurken and DAPI.
(TIF)

Figure S5 **Levels of Grk protein are unaffected in** ***heph*** **mutant ovaries.** Western blot showing relative levels of Grk and Heph in wild-type Oregon-R (OrR) ovarian extracts and in ovarian extracts prepared from *heph* germline clones. Tubulin was used as a loading control.
(TIF)

Table S1 **16 trans-acting factors identified by GRNA affinity chromatography to associate with 5′ORF RNA.** Heph/PTB is highlighted in bold. Adapted from McDermott et al. [34].
(DOC)
