# Supplementary figures and images for: Drosophila Hephaestus/Polypyrimidine Tract Binding Protein Is Required for Dorso-Ventral Patterning and Regulation of Signalling between the Germline and Soma
Source: PLoS One. 2013 Jul 23;8(7):e69978. doi: 10.1371/journal.pone.0069978 (PMC3720928; doi:10.1371/journal.pone.0069978)

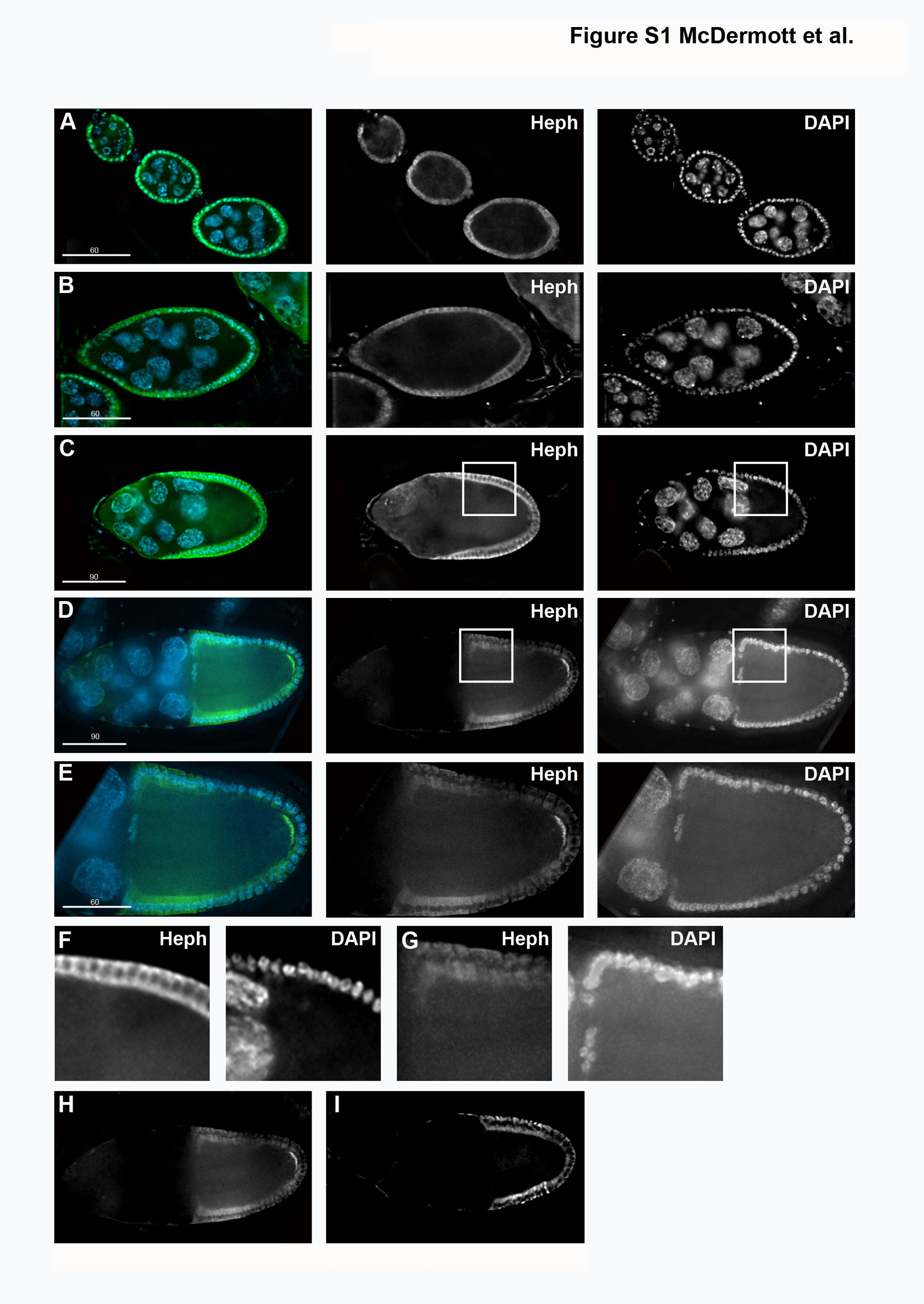

Supplement: Figure S1 — Distribution of Heph during Drosophila oogenesis. Stages 4–6 (A), stage 8 (B), stage 9 (C) and stage 10B (D and E) egg chambers stained with anti-Heph antibodies and DAPI. (E) is an enlargement of the oocyte shown in the egg chamber in (D). (F) is an enlargement of the dorso-anterior corner (white box) of the stage 9 oocyte in (C) whilst (G) is an enlargement of the dorso-anterior corner (white box) of the stage 10B oocyte in (D). Heph is detected in the cytoplasm of the follicular epithelial cells throughout oogenesis. In the germline, Heph is present throughout the oocyte cytoplasm with an accumulation at the posterior pole of the oocyte from around stage 9 of oogenesis onward (C–E). However, Heph does not accumulate at the dorso-anterior cap coincident with grk mRNA (C–G). Scale bars in A, B, and E represent 60 µm. Scale bars in C and D represent 90 µm. (H) is the same wild-type egg chamber shown in (D) for comparison with (I), which is a hephe1 germline clone egg chamber also stained with anti-Heph antibodies. Oocyte and nurse cell signals are lost in hephe1 mutant egg chambers, indicating that the signal is specific. (TIF) [file pone.0069978.s001.tif]

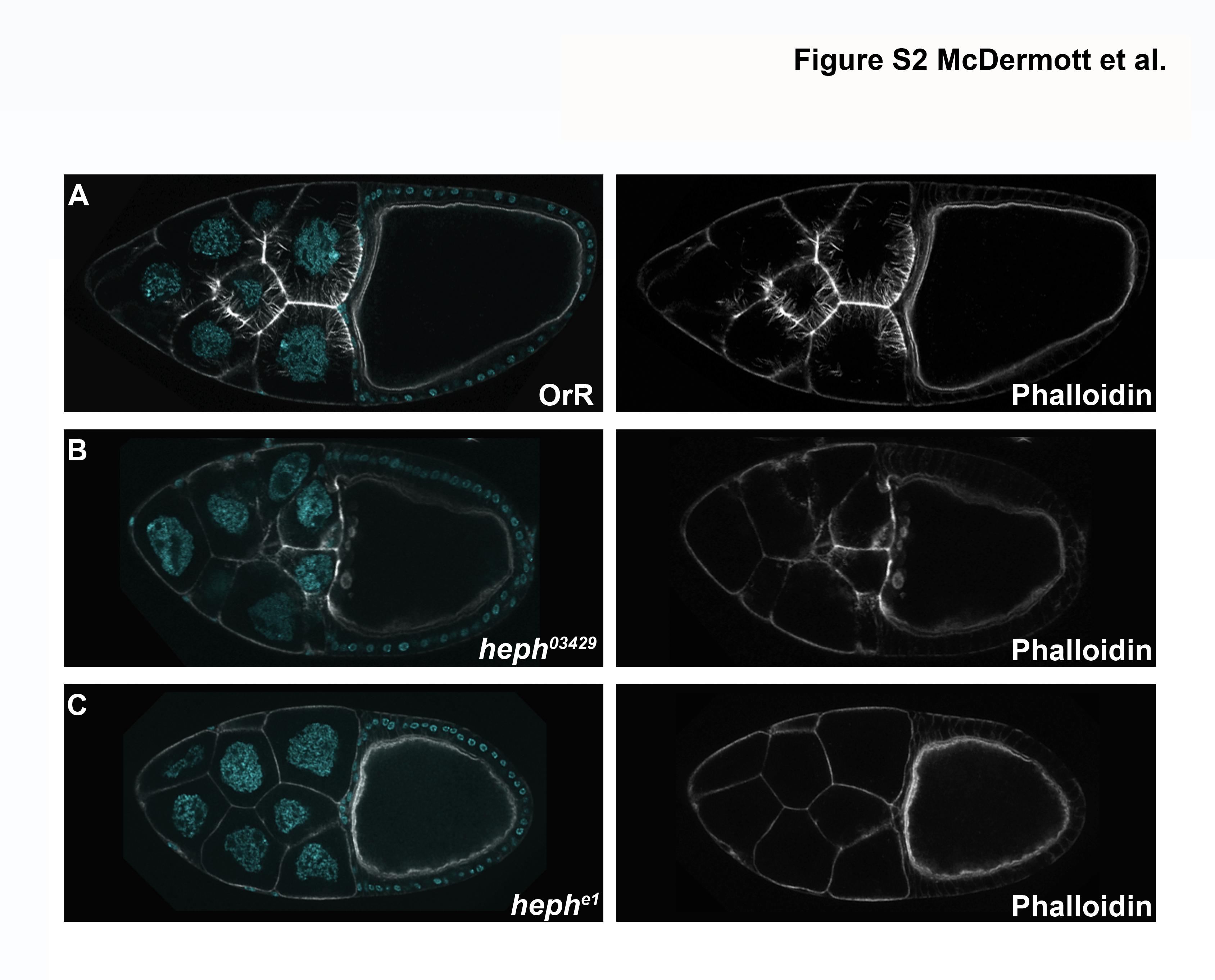

Supplement: Figure S2 — Actin structures are disrupted in heph03429 and hephe1 germline clone egg chambers. Wild-type Oregon-R (OrR) (A), heph03429 germline clone (B), and hephe1 germline clone egg chambers (C) were stained with Phalloidin to label F-actin. (TIF) [file pone.0069978.s002.tif]

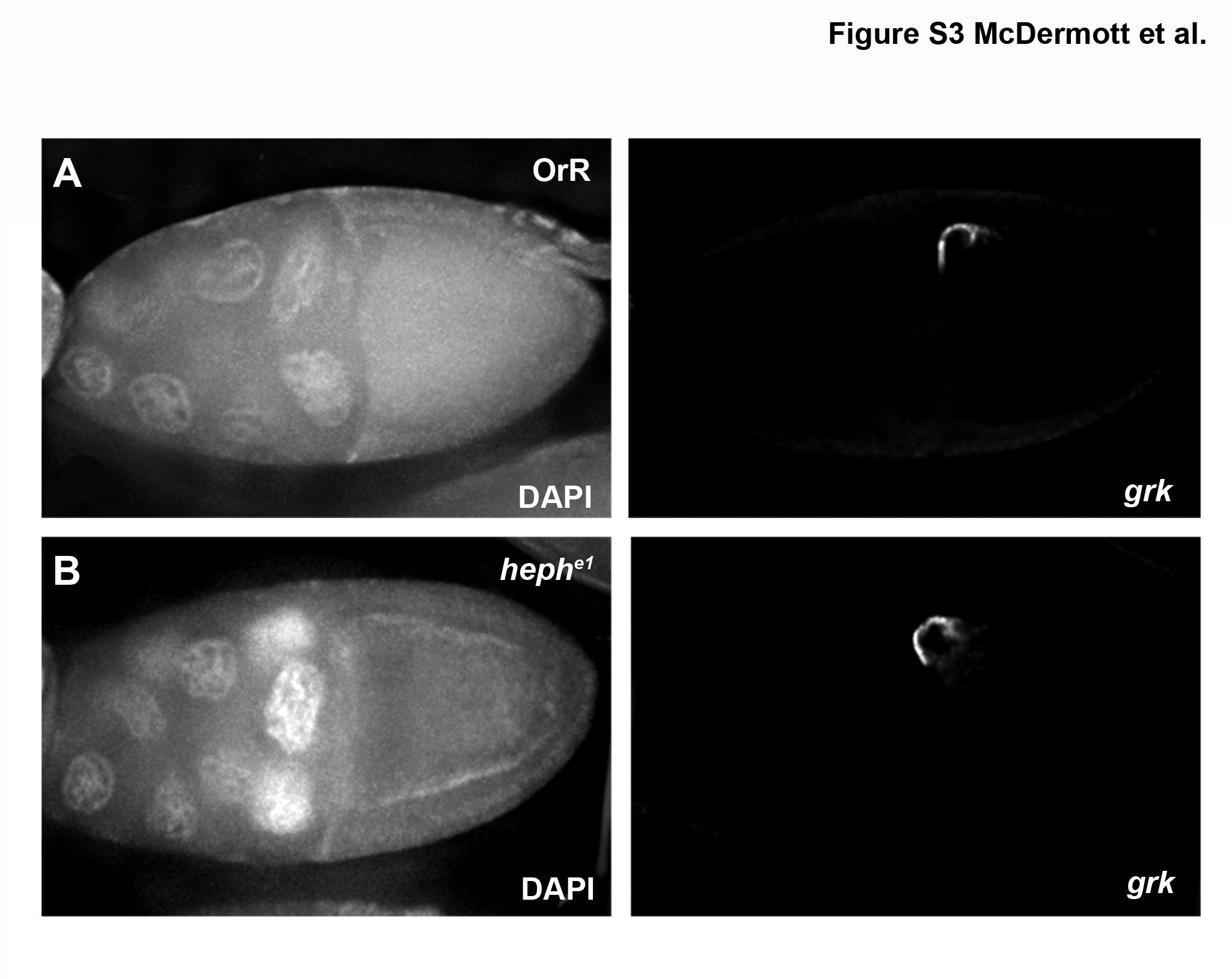

Supplement: Figure S3 — grk mRNA localization is not perturbed in stage 10 heph mutant oocytes. In situ hybridization using a grk was carried out on Oregon-R (OrR) (A), or hephe1 germline clone egg chambers (B). Egg chambers were also stained with DAPI to label DNA. (TIF) [file pone.0069978.s003.tif]

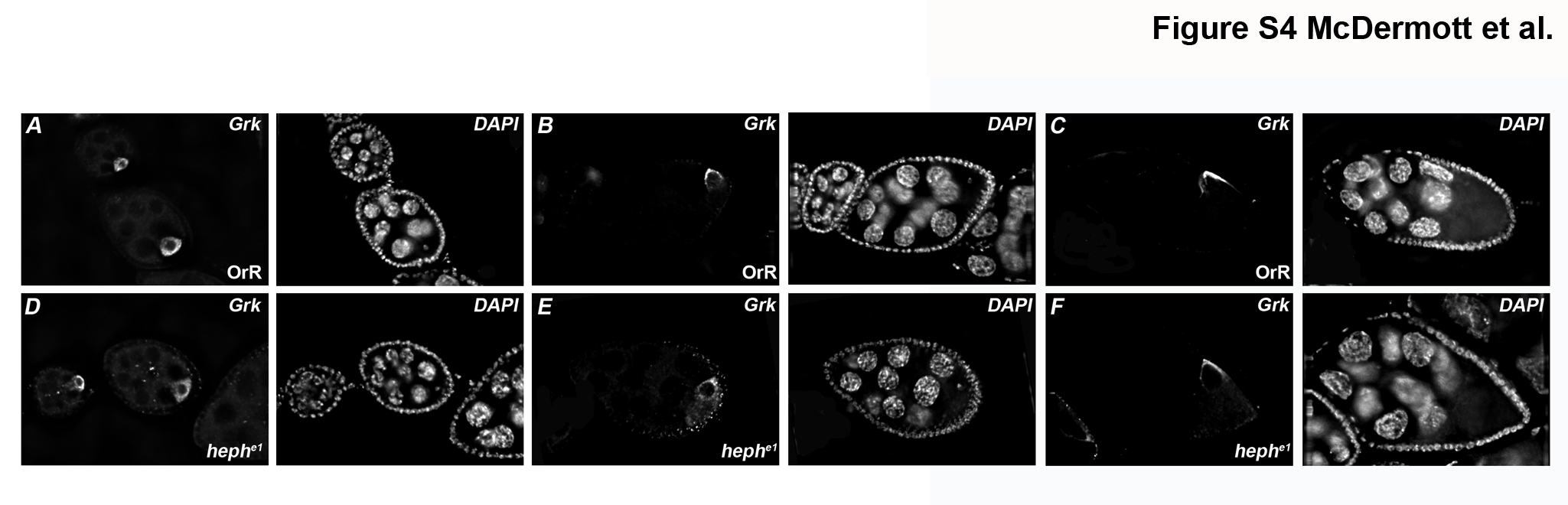

Supplement: Figure S4 — Grk protein localization in heph mutant stage 6 to stage 9 oocytes. Stage 6 (A), stage 8 (B), and stage 9 (C) wild-type Oregon-R (OrR) egg chambers, and stage 6 (D), stage 8 (E), and stage 9 (F) hephe1 germline clone egg chambers were stained with anti-Gurken and DAPI. (TIF) [file pone.0069978.s004.tif]

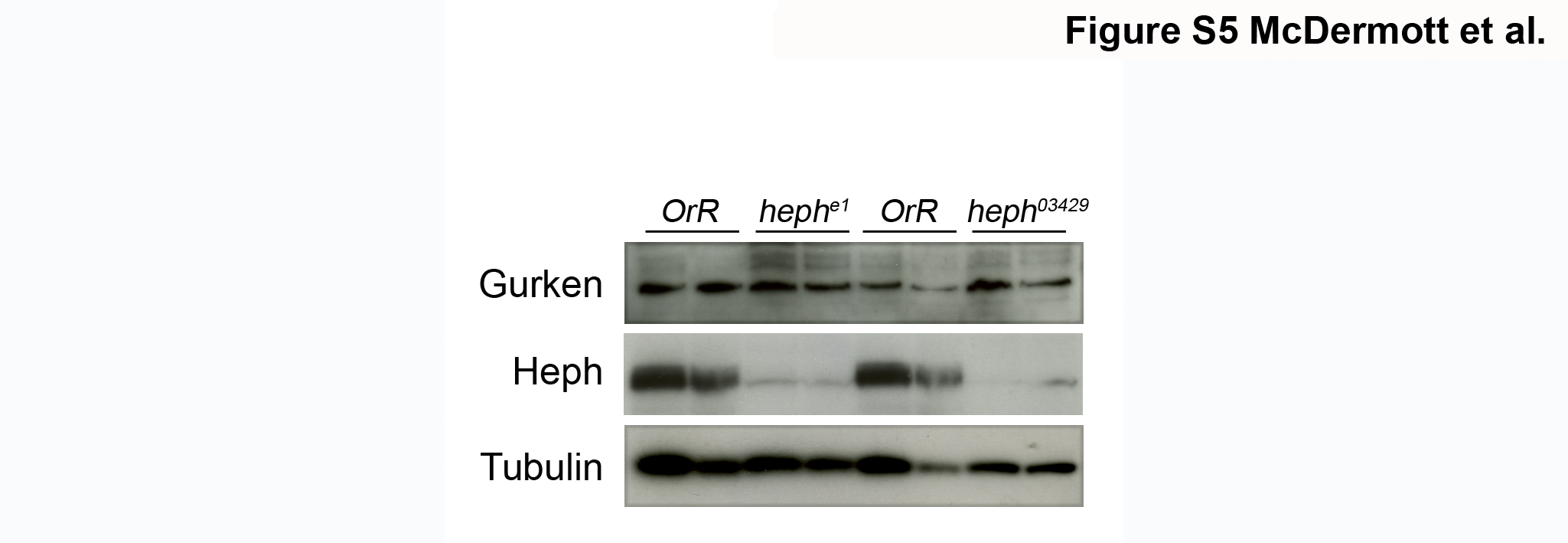

Supplement: Figure S5 — Levels of Grk protein are unaffected in heph mutant ovaries. Western blot showing relative levels of Grk and Heph in wild-type Oregon-R (OrR) ovarian extracts and in ovarian extracts prepared from heph germline clones. Tubulin was used as a loading control. (TIF) [file pone.0069978.s005.tif]
