# Supplementary material for: Drosophila Hephaestus/Polypyrimidine Tract Binding Protein Is Required for Dorso-Ventral Patterning and Regulation of Signalling between the Germline and Soma
Source: PLoS One. 2013 Jul 23;8(7):e69978. doi: 10.1371/journal.pone.0069978 (PMC3720928; doi:10.1371/journal.pone.0069978)
Supplement: Table S1 — 16 trans-acting factors identified by GRNA affinity chromatography to associate with 5′ORF RNA. Heph/PTB is highlighted in bold. Adapted from McDermott et al. [34]. (DOC) [file pone.0069978.s006.doc]

**Table S1**

| Function | Protein type | Protein name |
| --- | --- | --- |
| mRNA localization | RNA-binding protein | IGF-II mRNA-binding protein (Imp), Exuperentia (Exu) |
| mRNA localization and translational regulation | RNA-binding protein | Squid (Sqd), **Hephaestus (Heph)/Polypyrimidine tract binding protein (PTB)** |
| Translational regulation | RNA-binding protein | Dodeca satellite binding protein 1 (Dp1), Bicaudal C (BicC), Polyadenylate-binding protein (PABP) |
| Translational regulation | RNA helicase | Maternal expression at 31B (Me31B) |
| Other RNA processing | RNA-binding protein | Fibrillarin (Fib), CG6745 |
| Unknown function | RNA-binding protein | CG17838 Drosophila Syp |
| Unknown function | RNA helicase | CG5205 |
| Structural constituent of the ribosome | Ribosome | Ribosomal protein L14 (RpL14), Ribosomal protein S19 (RpS19) |
| Regulation of transcription | Transcription | Rigor mortis (Rig) |
| Other | Other | Deadhead (Dhd) |

16 trans-acting factors identified by GRNA affinity chromatography to associate with 5’ORF RNA. Heph/PTB is highlighted in bold. Adapted from McDermott et al. [34].
